# Gut microbiome as a potential mediator linking sexual behaviors to immune profiles in HIV-negative men who have sex with men: a multi-omics study

**DOI:** 10.3389/fimmu.2025.1659556

**Published:** 2025-10-16

**Authors:** Kangjie Li, Haijiao Zeng, Tian Liu, Siyan Huo, Cong Zhang, Wenlong Li, Jielian Deng, Xiaohua Zhong, Yi Tao, Bing Lin, Jiaxiu Liu, Biao Xie, Xiaoni Zhong

**Affiliations:** ^1^ School of Public Health, Research Center for Medicine and Social Development, Chongqing Medical University, Chongqing, China; ^2^ Department of Pharmacology and Toxicology, Medical College of Wisconsin, Milwaukee, WI, United States; ^3^ Daping Hospital, Army Medical University, Chongqing, China; ^4^ Sichuan International Studies University, Chongqing, China; ^5^ The First Affiliated Hospital of Chongqing Medical University, Chongqing, China; ^6^ College of Artificial Intelligence Medicine, Chongqing Medical University, Chongqing, China

**Keywords:** gut microbiome, sexual behaviors, men who have sex with men, immunology, causal mediation analysis

## Abstract

**Introduction:**

The effects of sexual behaviors on the gut microbiome and immune system in men who have sex with men (MSM) remain unclear. Here, we conducted a multi-omics study in MSM to investigate how sexual behaviors shape gut microbiome composition and immune profiles in this population. The interplay among high-risk sexual behaviors, gut microbiome, and systemic immune activation was also explored.

**Methods:**

HIV-negative MSM were enrolled in this study. Fecal samples were collected and subjected to 16S rRNA gene sequencing. Bulk and single-cell transcriptome sequencing of peripheral blood mononuclear cells (PBMCs) were performed to investigate the systemic immune profiles. Primary component analysis and spearman correlation analysis were used to assess the associations between gut microbiome and immune signatures. BayesPrism algorithm was applied to predict cellular composition and gene expression in individual cell types by integrating bulk RNA sequencing and sc-RNA sequencing. Causal mediation analysis evaluated the contribution of gut microbiome in linking sexual behaviors to immune outcomes.

**Results:**

The gut microbiome of HIV-negative MSM was dominated by *Segatella*. Receptive anal intercourse had the most significant impact on the gut microbiome, characterized by increased diversity, depletion of *Xylanibacter*, and enrichment of *Holdemania*. We also identified altered immune gene expression, an elevated CD8:CD4 ratio, distinctive CD4^+^ T cell communications, and higher expression of CXCR4 in CD4^+^ T cells in MSM engaged in receptive anal intercourse. Mediation analysis indicated that *Bilophila* potentially mediated the effects of receptive anal intercourse on CD4^+^ T cell proportions (*P* = 0.026). MSM exposed to group sex and illicit drug had elevated HIV susceptibility index, possibly mediated by *Bifidobacterium* (*P* = 0.012, *P* = 0.02 respectively).

**Conclusion:**

Our study indicates that gut microbiome partially mediates the immunomodulatory effects of sexual behaviors, providing mechanistic insights into HIV susceptibility. These findings underscore the gut-immune axis as a potential target for HIV prevention strategies in high-risk MSM.

## 1 Introduction

Dysbiosis of the gut microbiome has been implicated in promoting HIV infection (1). A longitudinal study revealed that pre-existing gut microbial differences were observed in patients with acute HIV infection compared to seronegative controls, highlighting the significance of the gut microbiome in enhancing HIV susceptibility (2). The gut microbiome in HIV-infected individuals is typically characterized by decreased alpha diversity (3–7), enrichment of *Prevotella*, and depletion of *Bacteroides (* 3, 8). However, studies examining the relationship between HIV status and gut microbiome diversity have yielded inconsistent results, likely due to the influence of sample size, sexual practices, substance use, and antiretroviral therapy (9–15). Sexual orientation is particularly influential, as MSM account for a large proportion of HIV cases (16). Findings in seronegative controls without matching for MSM status fail to accurately reflect the effects of HIV infection on the human gut microbiome. A recent well-designed study, which matched sexual orientation and other confounding factors, demonstrated that MSM exhibited distinct microbial characteristics independent of HIV status (6). Compared with non-MSM, the gut microbiome of MSM showed higher diversity and was dominated by the *Prevotella* genus (17, 18). The MSM status even had a more profound impact on the composition of the gut microbiome than HIV status (19, 20). Among MSM, receptive anal intercourse has a notable impact on gut microbial communities (6, 14, 21, 22). The number of male partners is also significantly associated with gut microbiome composition (17). However, these studies were primarily designed to identify the influence of HIV status or MSM status on the gut microbiome, and the true effects of sexual behaviors could be confounded by HIV status.

Studies have indicated that gut microbiome dysbiosis can drive mucosal immune activation, resulting in an increased risk of HIV infection. In HIV-negative MSM, gut microbiota appears to drive the influx of CCR5^+^ CD4^+^ T cells, which are preferentially targeted by HIV, into the gut (23). Additionally, gut microbiota correlates with inflammatory factors and microbial translocation factors in the plasma, such as C-reactive protein, sCD14, and LPS Binding Protein (LBP) (8, 24). Regardless of HIV status, MSM present higher levels of inflammatory factors, which are linked to gut microbiome species (25). Moreover, gut microbiota from HIV-negative MSM has been shown to activate immune responses in gnotobiotic mice, increasing the frequency of gut CD69^+^ and CD103^+^ T cells and enhancing HIV infection (26). Notably, *Holdemanella biformis* upregulates the expression of CCR5, a co-receptor of HIV-1, in CD4^+^ T cells, facilitating HIV infection (27). While these studies have advanced our understanding, they mainly involve animal models and *in vitro* experiments, limiting insights into the comprehensive spectrum of gut microbiome-immune interactions in MSM.

It is well established that receptive anal intercourse and other sexual behaviors elevate the risk of HIV acquisition in MSM (28). However, the interactions among high-risk sexual behaviors, gut microbiome, and immune signatures are not yet fully understood. We hypothesize that gut microbiome dysbiosis following high-risk sexual behavior stimulates immune activation and increases the risk of HIV infection. In the present study, we collected fecal samples from HIV-negative MSM and performed 16S rRNA gene sequencing to characterize the gut microbiome composition. We aimed to assess the impact of sexual behaviors on the gut microbiome and to identify the specific behavior with the most significant effects. To further elucidate the associations between the gut microbiome and systemic immune signatures, peripheral blood mononuclear cells (PBMCs) from these participants were isolated, and both bulk sequencing and single-cell RNA sequencing were conducted. In addition, we performed causal mediation analysis to investigate the role of gut microbiome in linking sexual behaviors and systemic immune signatures. This multi-omics analysis provides novel insights into the intricate interactions among sexual behaviors, gut microbiome, and immune systems.

## 2 Materials and methods

### 2.1 Study participant

Men who have sex with men were recruited from Chongqing city, and the recruitment advertisement was spread with the help of local MSM community members. Recruitment and samples collection were conducted between Oct 2023 and Nov 2023. Participants who engaged in anal intercourse with men within 6 months before recruitment were enrolled for this study. Sexual behaviors were self-reported. Volunteers were excluded when they met one of the following criteria: 1) under 18 years of age, 2) vegetarian, 3) being infected with HIV, HBV or HCV, 4) history of the heavy gastrointestinal diseases including ulcers, cancers and other inflammatory diseases, 5) history of any gastrointestinal operation, 6) history of the metabolic diseases including diabetes, hypertension and hyperlipemia, 7) history of any antibiotic or prebiotic use 3 months before recruitment. In this study, HIV antibody and HCV antibody in the plasma were tested by ELISA method, and HBsAg in the blood was tested by colloidal gold method.

### 2.2 Sample collection

Fecal samples were collected by the participants with a sterile scoop and stored in a fecal DNA storage tube at the room temperature, which could reduce the degradation of the fecal DNA. Fecal samples were delivered to the lab within 24 hours after the visit, and were stored at the -80 °C, awaiting DNA extraction for the 16S rRNA gene sequencing. Two tubes of blood were taken from participants by the doctor, 5 ml for each tube. Tubes contained EDTA for anticoagulation and stored at 4 °C. All blood samples were sent for PBMC isolation within 2 hours.

### 2.3 PBMCs isolation

PBMCs isolation was conducted by density gradient centrifugation using Ficoll-Paque Premium (1.077 g/ml, GE Healthcare) according to the manufacturer’s instructions. Briefly, the whole blood was diluted with PBS at 1:1 ratio. The diluted blood was layered carefully onto the Ficoll liquid in a 15 ml centrifuge tube, and centrifuged at 400g and 18 to 20 °C for 30 minutes without breaks. The PBMCs layer was then transferred to a 15 ml sterile centrifuge tube and mixed with 3 volumes of PBS for washing, following centrifuging at 400g and 18 to 20 °C for 10 minutes. Repeat the washing once again and discard the supernatant. PBMCs were cryopreserved in cell freezing medium composed of 90% fetal blood serum and 10% DMSO, and stored at -80 °C.

### 2.4 Fecal DNA extraction and 16S rRNA gene sequencing analysis

Total fecal DNA was extracted with the following steps. 200 mg fecal sample was transferred to the centrifuge tube with grinding beads. Add 1 ml buffer ATL/PVP-10, grinding the sample with the grinding machine and incubate at 65 °C for 20 minutes. The grinded sample was then centrifuged at 14000g for 5 minutes, and the supernatant was transferred to a new tube with 0.6 ul buffer PCI and mixed thoroughly by vortex for 15s. The sample was then centrifuged at 18213g for 10 minutes. Transfer the supernatant to deep well plate with 600 ul magnetic beads binding solution containing 20 ul proteinase K, 5 ul RNase and 100 ul elution buffer. The deep well plate was then transferred to the proper place of the machine and the corresponding program started in Kingfisher. After the program finished, the extracted DNA was transferred to a new 1.5 ml centrifuge tube.

The V3-V4 regions of the 16S rRNA gene were amplified using polymerase chain reaction (PCR) with the barcoded primers (338F-806R, FWD: ACTCCTACGGGAGGCAGCAG, REV: GGACTACHVGGGTWTCTAAT). Amplified DNA was then quantified and equal amounts of amplicons were pooled and sequenced based on the DNBSEQ G400 PE300 platform. Raw sequence data were filtered and reads that were low quality and contaminated with adapter sequences were removed. Tags were clustered to generate OTU (operational taxonomic unit) with USEARCH (v7.0.1090) according to 97% sequence similarity. And then the OTU representative sequences were aligned against the RDP database (Release 19) for taxonomic annotation by RDP classifier software (v2.14).

Alpha diversity indexes were calculated and the beta diversity was evaluated based on Bray-Curtis distances using R package vegan v2.6–4 at the OTU level. Wilcoxon rank-sum test or Kruskal-wallis test was conducted to compare the differences of alpha diversity among groups. For beta diversity, principal co-ordinates analysis (PCoA) was utilized to evaluate the global microbial composition. Analysis of similarities (ANOISM) and PERMANOVA statistical test (R function ‘adonis’) were performed to compare the differences of beta diversity between groups. To identify differentially abundant genus between groups, genera that presented in fewer than 20% samples were filtered out. Wilcoxon rank-sum test was conducted to analysis the statistic differences of the relative abundance of genera. *P* < 0.05 was considered significant.

### 2.5 Bulk RNA sequencing analysis of PBMCs

Total RNA of PBMCs was extracted using TRIzol-chloroform according to manufacturing instruction. Briefly, TRIzol lysis buffer was added into the cell sample tube and centrifuged at 12000g for 5 minutes in 4°C. Transfer the supernatant to centrifuge tube with 300 ul Chloroform/isoamyl-alcohol (24:1) and mix thoroughly by upside down violent shaking. Centrifuge the mixture at 12000g for 8 minutes in 4°C. Transfer the supernatant to the 1.5ml centrifuge tube, add 2/3 volume of isopropyl-alcohol, invert and mix gently and place in -20°C for more than 2 hours. After that, centrifuge at 17500g for 25 minutes in 4°C and discard the supernatant. The precipitation was washed with 75% ethanol twice and then dissolved in RNase-free water. Library construction was performed following these steps. Total RNA was denatured to open their secondary structure, and mRNA was enriched by oligo (dT)-attached magnetic beads. The mRNA was then fragmented and subjected to double-stranded cDNA synthesis. The PCR reaction system was then set up to amplify the product. The PCR product was quality controlled and sequenced on DNBSEQ G400 SE50 platform.

The sequencing data was filtered with SOAPnuke software (v1.5.6). Low-quality and contaminated reads were removed. Reads were then aligned to the transcriptome by Bowtie2 (2.3.4.3). Expression level of gene was calculated by RSEM (v1.3.1). Differential expression analysis was performed using the DEGseq with Q value ≤ 0.05 and Log2FC ≥ 1. To take insight to the change of phenotype, GO and KEGG enrichment analysis of differently expressed genes was performed by clusterProfiler R package (v4.8.3).

### 2.6 Single-cell RNA sequencing analysis of PBMCs

The 10x Genomics protocol was applied to prepare the libraries of single-cell cDNA. The cellular suspension was stained with 0.4% trypan blue to access cell viability under microscopic observation, and cells with greater than 80% viability were qualified for next library construction. Single-cell suspension was then partitioned into GEMs (Gel Beads in Emulsions) in the automated Chromium Controller, and then mRNAs were reverse transcribed into cDNAs. After the reaction finished, the cDNA was purified and amplified in the PCR reaction system. Then equal amount of cDNAs were subjected to fragmentation, end repair and addition of “A” base at the 3’-end of each strand. The products were then subjected to adaptor ligation and amplified through PCR. Finally, the cDNAs were denatured and the products were cycled to produce single-strand circle DNA molecules. The prepared DNA libraries were sequenced on DNASEQ G400 PE100 platform.

The raw gene expression matrix generated from each sample were aggregated using CellRanger (v5.0.1) provided on 10x genomics website. Downstream single-cell analysis was done using the R package Seurat (v5.0.1). Quality control was applied to cells based on the number of detected genes, number of UMIs and proportion of mitochondrial reads per cell. Cells with less than 200 detected genes or with more than 95% of the maximum genes (2000) were filtered out. Besides, cells with more than 5% percentage of mitochondrial counts were also removed. Moreover, we excluded cells with more than 10000 UMIs. The final matrix was then normalized and subsequent principal component analysis was conducted using the 2000 highly variable features. Number of the principal components was selected based on the elbow plot. Batches among samples were integrated using the harmony correction algorithm (v1.2.0). R package clustree (v0.5.1) was used to visualize the clustering at different resolution, and the best resolution parameter was chosen based on the clustering tree. Manual cluster annotation was performed based on known markers according to Cellmarker database (v2.0) and published literatures. U-MAP was used for two-dimensional visualization of the clusters.

Differential expression analysis between clusters was conducted by Wilcoxon rank-sum test in the R package Seurat with the following parameters: min.pct = 0.25, logfc.threshold = 0.2. Cell-cell communication analysis was performed using R package CellChat (v1.6.1) with default parameters. R package scAB (1.0.0) was applied to analyze the association between phenotype and single cells with default parameters.

### 2.7 Random forest machine learning approach

Random forest model was constructed by R package randomForest (v4.7-1.1). As the number of MSM who engaged RAI was nearly twice that of MSM never engaged in RAI, the dataset was considered as “class imbalance”. Over-sampling was conducted to solve the imbalance. The dataset was randomly divided into training set and test set; 70% samples were used as the training set and remaining 30% as the test set. An internal five-fold cross-validation was applied in the training set to select the best hyperparameters. The final model was created with the following hyperparameters: ntree=10 and mtry=5. The area under the receiver operating characteristic curve (AUC) was accessed to evaluate the performance of the model in the test set.

### 2.8 Correlation analysis between immune signatures and gut microbiotas

Gut microbiota taxa and immune genes which were identified to associate with receptive anal intercourse were included in the correlation analysis. To address high-dimensionality challenges, principal component analysis (PCA) was independently performed on the gut microbiota abundance matrix and immune gene expression dataset. The first “N” principal components explaining 80% cumulative variance contribution were selected for downstream analyses. The associations were then assessed using Spearman’s rank correlation coefficients between gut microbial and immune principal components. Statistical significance was determined at a threshold of *P* < 0.05.

### 2.9 BayesPrism analysis

BayesPrism algorithm was performed to deconvolute bulk RNA sequencing data into distinct cell types (‘BayesPrism’ R package, v2.2). This approach aims to predict cellular composition and gene expression in individual cell types from bulk RNA-seq using scRNA-seq as prior information (29). Our four-sample annotated single-cell RNA sequencing datasets were used as the reference datasets to deconvolute the proportion of different cell types across the 98 samples. The analysis was performed using the algorithm’s default parameters.

### 2.10 HIV susceptibility index calculation

To explore the association between gut microbiotas and HIV susceptibility, we constructed a novel index which we called “HIV susceptibility index”. HIV susceptibility index (‘HIV-SI’) was calculated as the log ratio of geometric means of four genes in the CD4^+^ T cells, including CCR5, CCXR4, ITGA4, and ITGB7. The equation was as follows:


$$HIV\_SI=\log_{10}\sqrt[{4}]{CXCR4*CCR5*ITGA4*ITGB7}$$

where CXCR4, CCR5, ITGA4 and ITGB7 represent the expression levels in CD4 T cells based on the Bayesprism algorithm. CCR5 and CXCR4 serve as the primary co-receptors of HIV-1 viral entry into host cells. ITGA4 and ITGB7 encode the units of α4β7 integrin which mediates the lymphocytes immigrating to the intestinal lamina propria (30). The HIV-SI mechanistically quantifies the propensity of CD4^+^ T cells to undergo gut-homing and subsequent HIV-1 infection.

### 2.11 Causal mediation analysis

The ‘mediation’ R package (v4.5.0) was utilized to perform the causal mediation analysis using the mediate function. Bootstrapping was used to test the significance of the indirect effects with 999 Monte Carlo draws. Six sexual behaviors were selected as the exposure variables, including receptive anal intercourse, number of male sex partners, HIV^+^ partners, group sex with man, illicit drug use and STDs. CD4^+^ T cell proportions, CD8^+^ T cell T cell proportions, CD4:CD8 ratio and HIV-SI served as the outcome variables. Gut microbiome which was significantly associated with both the exposure variables and the outcome variables were selected as the mediators. It’s important to mention that age and BMI were included in mediation analysis to control the confounding effects.

### 2.12 Statistical analysis

All analysis were conducted using R software (v4.3.0). Statistical significance was defined as a two-sided *P* value less than 0.05.

## 3 Results

### 3.1 Demographic and sexual behavior characteristics of participants

To investigate the sexual behavior associated gut microbial communities without the additional influences of HIV infection, we recruited HIV-negative MSM for this study. We initially enrolled 100 eligible participants, of which five fecal samples and two PBMC samples failed the DNA quality assessment respectively. Consequently, the gut microbiota analysis comprised 95 samples (95% of the total cohort), while the bulk omics analysis included 98 samples (98%). All participants were residents of Chongqing city in China. None of participants reported having used antibiotic or prebiotic within 3 months prior to the investigation. The age, body mass index (BMI) and sexual behaviors of these participants included in the gut microbiome analysis were stated in the **Table 1**. Sixty-two MSM reported receptive anal intercourse with men within the past 6 months (RAI subgroup), while 33 MSM engaged only in insertive anal intercourse (nonRAI subgroup). Only one MSM reported having an HIV-positive male partner. Seven participants self-reported a diagnosis of sexually transmitted diseases (STDs) within the past six months, which was corroborated by blood testing results for treponema pallidum antibody and HBsAg.

**Table 1:** Demographic and sexual behavior characteristics of enrolled men who have sex with men in the 16S rRNA gene sequencing.

Demographic and sexual behavior parameter	N (95)
Age	
< 20	3 (0%)
20 ~	44 (39.4%)
30 ~	32 (36.4%)
40 ~	16 (24.2%)
BMI	
< 18	16 (9.1%)
18 ~ 24	63 (78.8%)
24 ~ 28	4 (6.1%)
> 28	12 (6.1%)
Receptive anal intercourse	
nonRAI	33 (34.7%)
RAI	62 (48.4%)
Number of male partners	
<= 2	51 (53.7%)
> 2	44 (46.3%)
Condom	
Never	6 (6.3%)
Sometimes	18 (18.9%)
Always	71 (74.7%)
HIV^+^ partner (including male and female)	
None	85 (89.5%)
Unknown	9 (9.5%)
Yes	1 (1.0%)
Commercial sex with men	
No	89 (93.7%)
Yes	6 (6.3%)
Group sex with men	
No	87 (91.6%)
Yes	8 (8.4%)
Sexually transmitted diseases	
No	88 (92.6%)
Yes	7 (7.4%)
Illicit drug	
No	88 (92.6%)
Yes	7 (7.4%)

### 3.2 Sexual behaviors are linked with gut microbial community shift

**Figure 1:**
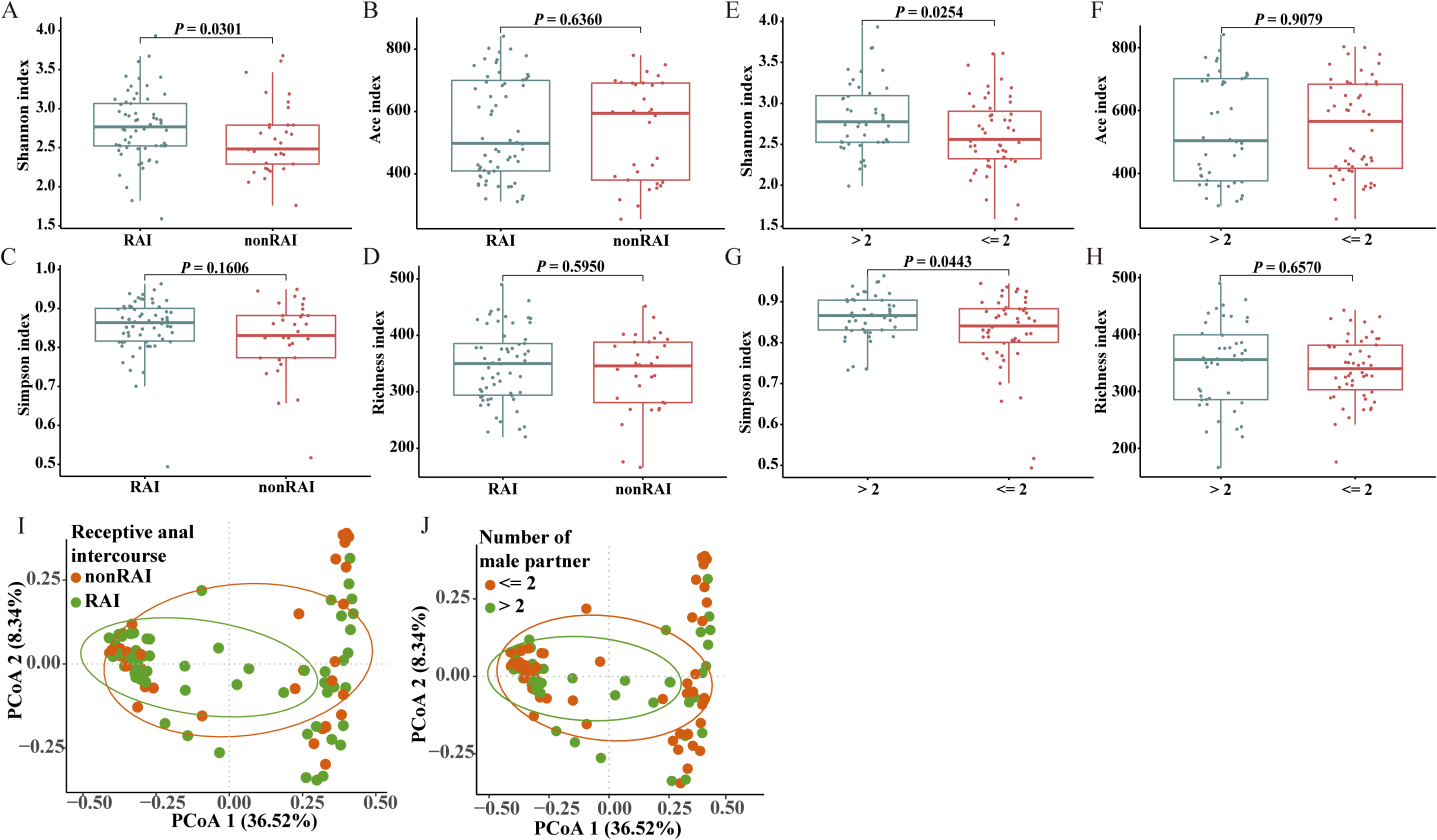
Comparisons of gut microbiome diversities across different sexual behaviors. **(A-D)** Box plot depicting alpha diversity analysis of MSM in RAI subgroup compared to MSM in nonRAI subgroup based on Wilcoxon rank-sum test. **(E-H)** Box plot depicting alpha diversity analysis of MSM with > 2 male sex partners compared to MSM with<= 2 male sex partners based on Wilcoxon rank-sum test. **(I)** Dot plot for results of principal-coordinate analysis (PCoA) using Bray-Curtis distance between RAI subgroup and nonRAI subgroup. ANOISM test showed significant differences of beta diversity between groups (R = 0.0797, P = 0.0194), and PERMANOVA test depicted marginally statistical significance of the beta diversity (R2 = 0.0181, P = 0.0882). **(J)** Dot plot for results of PCoA using Bray-Curtis distance between MSM with > 2 male sex partners and MSM with<= 2 male sex partners subgroups. ANOISM test and PERMANOVA test showed insignificant differences of beta diversity between groups (R = -0.0078, P = 0.6486; R2 = 0.0107, P = 0.3517). See also **Supplementary Figure S1**.

Four indices were calculated to indicate the alpha diversity of gut microbiome in each individual sample, including Shannon index, Simpson index, Ace index, and Richness index. Significant alpha diversity differences were observed across multiple sexual behaviors, including anal sex role, number of male partners, having sex with HIV-positive partners, group sex with man, diagnosis of STDs, and illicit drug use (**Figure 1**, see also in **Supplementary Figure S1**). Comparison of Shannon index revealed higher microbiota diversity in MSM of RAI subgroup than nonRAI subgroup (*P* = 0.0301). MSM with more than two male sexual partners showed significantly higher gut microbiota diversity compared to those with two or fewer male partners, as evidenced by elevated Shannon (*P* = 0.0254) and Simpson (*P* = 0.0443) indices. The Simpson index of MSM who never had sex with HIV-positive partners was significantly lower than those who were unaware of their sexual partner’s HIV infection status (*P* = 0.0207), indicating lower diversity in the former subgroup. Participants who never engaged in group sex with man exhibited significantly higher Richness and Ace indices than those who had group sex with man (*P* = 0.0072, *P* = 0.0466 respectively). In addition, participants with diagnosis of STDs exhibited higher richness (*P* = 0.0338), and MSM with the history of illicit drug use had increased gut microbiome diversity as evidenced by increased Simpson index (*P* = 0.0249). While according to our results, condom use and commercial sex with man showed no associations with alpha diversity (*P* > 0.05).

For the beta diversity of the gut microbiome, Bray-Curtis distances were calculated based on the abundance at the OTU level (**Figure 1**, **Supplementary Figure S1**). ANOSIM analysis revealed significant differences in beta diversity between the RAI and nonRAI subgroups (R = 0.0797, *P* = 0.0194). We also performed PERMANOVA test to explore the differences of beta diversity between groups, which revealed a marginally significant difference between RAI and nonRAI subgroups (R^2^ = 0.0181, *P* = 0.0882). The result suggested a potential trend toward microbial community dissimilarity. Furthermore, other sexual behaviors, including condom use, sexually transmitted infections, and the number of male sex partners, were also evaluated to investigate their associations between gut microbiome structure. However, we did not observe any significant associations between these sexual behaviors and the beta diversity of gut microbiome. Overall, these results indicated that the dysbiosis of the gut microbiome in HIV-negative MSM was primarily associated with receptive anal intercourse.

**Figure 2:**
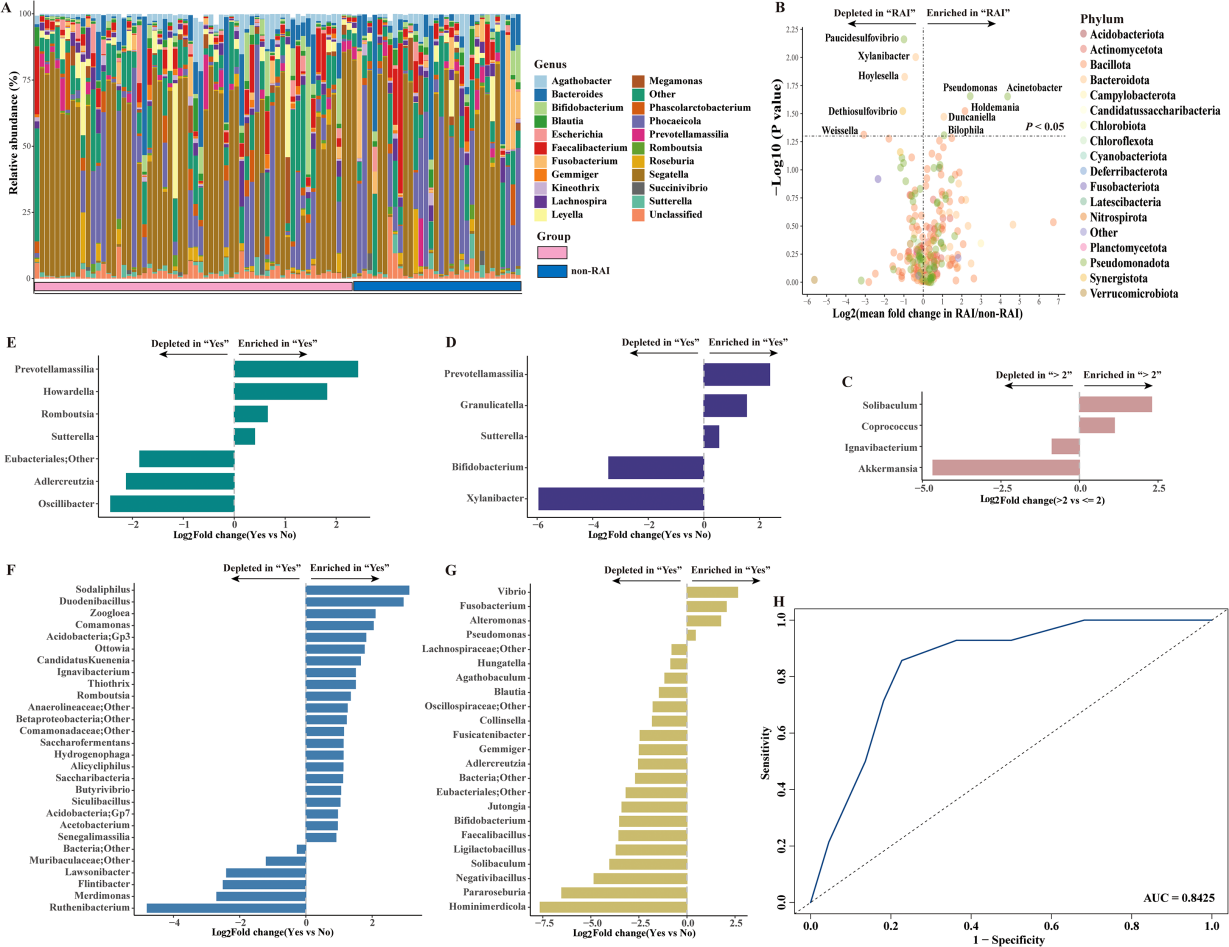
Spectrum of gut microbiome in HIV-negative MSM and the gut microbiome alterations across different sexual behaviors at genus level. **(A)** Bar plot showing the composition of gut microbiome in HIV-negative MSM at the genus level. **(B)** Volcano plot depicting gut microbiome in differential abundance between RAI subgroup and nonRAI subgroup. **(C)** Bar plot showing differentially abundant genera in MSM with > 2 male sex partners vs MSM with<= 2 male sex partners. **(D)** Bar plot showing differentially abundant genera in MSM reported illicit drug use vs MSM reported no history of illicit drug use. **(E)** Bar plot showing differentially abundant genera in MSM who had sex with undocumented HIV status man vs MSM who had sex with HIV-negative man. **(F)** Bar plot showing differentially abundant genera in MSM diagnosed with STDs vs MSM without STDs. **(G)** Bar plot showing differentially abundant genera in MSM who engaged in group sex vs MSM who never engaged in group sex. **(H)** Receiving occupational curve based on random forest model for classification of receptive anal intercourse status (RAI vs nonRAI). A good performance was achieved, with AUC of 0.8425 in test set.

We next compared the relative abundance of gut microbiome at genus level across different sexual behavior subgroups. At the genus level, *Segatella* was the predominant community in MSM, with a relative abundance of 39% and 30% in the RAI and nonRAI subgroups, respectively (**Figure 2A**). *Phocaeicola* was the second most dominant gut bacterial taxon, with a relative abundance exceeding 10% in both groups. Among the top five genera, *Segatella*, *Phocaeicola*, *Bacteroides*, and *Leyella* were part of the *Bacteroidota* phylum, while *Faecalibacterium* belonged to the *Bacillota* phylum.

Ten bacterial taxa were found to be significantly different between the RAI and nonRAI subgroups (**Figure 2B**). *Pseudomonas*, *Acinetobacter*, *Holdemania*, *Duncaniella*, and *Bilophila* were enriched in MSM with RAI, whereas *Paucidesulfovibrio*, *Xylanibacter*, *Hoylesella*, *Dethiosulfovibrio*, and *Weissella* decreased. Among these taxa, three bacteria were members of the *Bacteroidota* phylum (*Xylanibacter*, *Hoylesella*, and *Duncaniella*), four bacteria were members of the *Pseudomonadota* phylum (*Pseudomonas*, *Paucidesulfovibrio*, and *Acinetobacter*), and *Holdemania* and *Weissella* belonged to the *Bacillota* phylum. Compared to MSM with<= 2 male partners, we found an enrichment of *Solibaculum* and *Coprococcus*, coupled with depletion of *Ignavibacterium* and *Akkermansia* in those with > 2 male partners (**Figure 2C**). Illicit drug use showed significant effects on gut microbiome shift, demonstrated by enrichment of *Prevotellamassilia*, *Granulicatella*, and *Sutterella* and depletion of *Bifidobacterium*, and *Xylanibacter* in MSM reported history of illicit drug use (**Figure 2D**). Notably, the relative abundance of *Prevotellamassilia* also increased in MSM who were unaware of the HIV status of their sex partners (**Figure 2E**). Besides, twenty-eight genera were significantly differed between MSM with diagnosis of STDs and those without STDs, including enrichment of *Sodaliphilus* and depletion of *Ruthenibacterium* (**Figure 2F**). Moreover, we also found that MSM who conducted group sex with man was characterized by significantly higher abundance of *Vibrio*, *Fusobacterium*, *Alteromonas* and *Pseudomonas* (**Figure 2G**). Conversely, the abundance of *Hominimerdicola*, *Pararoseburia* and *Ligilactobacillus* were higher in those who never conducted group sex with man.

Given the fact that anal sex role exerted the most impact on gut microbiome community, we further applied a random forest model to assess the associations between gut microbiome and anal sex roles. The ten differentially abundant genera were enrolled in the model. The model achieved good microbiome-based classification capability, with an AUC of 0.8425 in the test set (**Figure 2H**). Collectively, our results demonstrated the important role of sexual behaviors in shaping gut microbiome community, with a pronounced effect observed for receptive anal intercourse.

### 3.3 Receptive anal intercourse interrupts immune homeostasis

**Figure 3:**
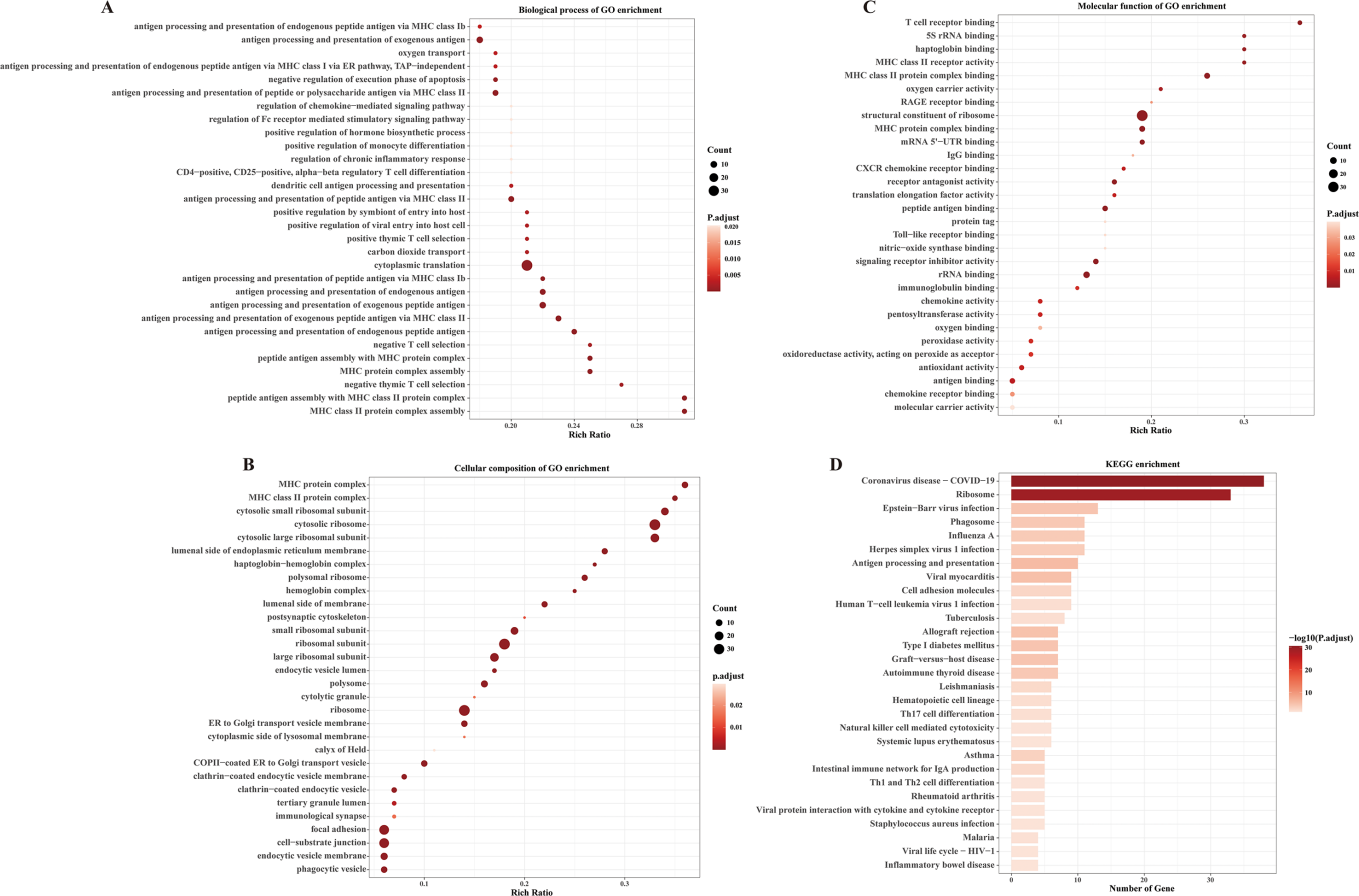
Receptive anal intercourse exerts significant influences on immunologic signatures in MSM. **(A)** Dot plot shows the enriched top 30 biological process of GO terms. **(B)** Dot plot shows the enriched top 30 cellular composition of GO terms. **(C)** Dot plot shows the enriched top 30 molecular functions of GO terms. **(D)** Bar plot shows the enriched KEGG pathways of the differentially expressed genes.

We further investigated whether receptive anal intercourse stimulated PBMCs activation through bulk RNA-seq analysis. The demographic information of these participants was stated in the **Supplementary Table S1**. Compared to MSM who never conducted receptive anal intercourse, 143 genes were upregulated and only one gene was downregulated in MSM who conducted receptive anal intercourse (**Supplementary Table S2**). Specifically, *FCGR3B* was downregulated in the RAI subgroup. Notably, several immune-related genes were significantly upregulated in the RAI subgroup, including *CXCR4*, *CXCL10*, *CCL5*, *FCER1G*, *CD52*, and *CLEC12A*. What worth of noting is that *CXCR4* is an important coreceptor for HIV infection, and its increased expression in the RAI subgroup suggests a higher HIV susceptibility in this subgroup. KEGG and GO enrichment analysis provided further insights into immunologic functions (**Figures 3A–D**). As expected, these differentially expressed genes were mainly enriched in KEGG pathways related to viral protein interaction with cytokine and cytokine receptor, viral life cycle—HIV-1, Epstein-Barr virus infection, Herpes simplex virus 1 infection, Th17 cell differentiation and natural killer cell mediated cytotoxicity. The biological process analysis of GO showed enrichment in regulation of viral entry into host cell, antigen processing and presentation, and T cell differentiation. Meanwhile, chemokine receptor binding, chemokine activity, immunoglobulin binding, and CXCR chemokine receptor binding were enriched in the molecular function analysis of GO. In the cellular component analysis of GO, immunological synapse and other immunology related process were enriched. These data suggest that receptive anal intercourse drives the activation of PBMCs in MSM, which is likely to increase the susceptibility to HIV infection.

### 3.4 Systematic immune signatures are closely related to gut microbiome in MSM

**Figure 4:**
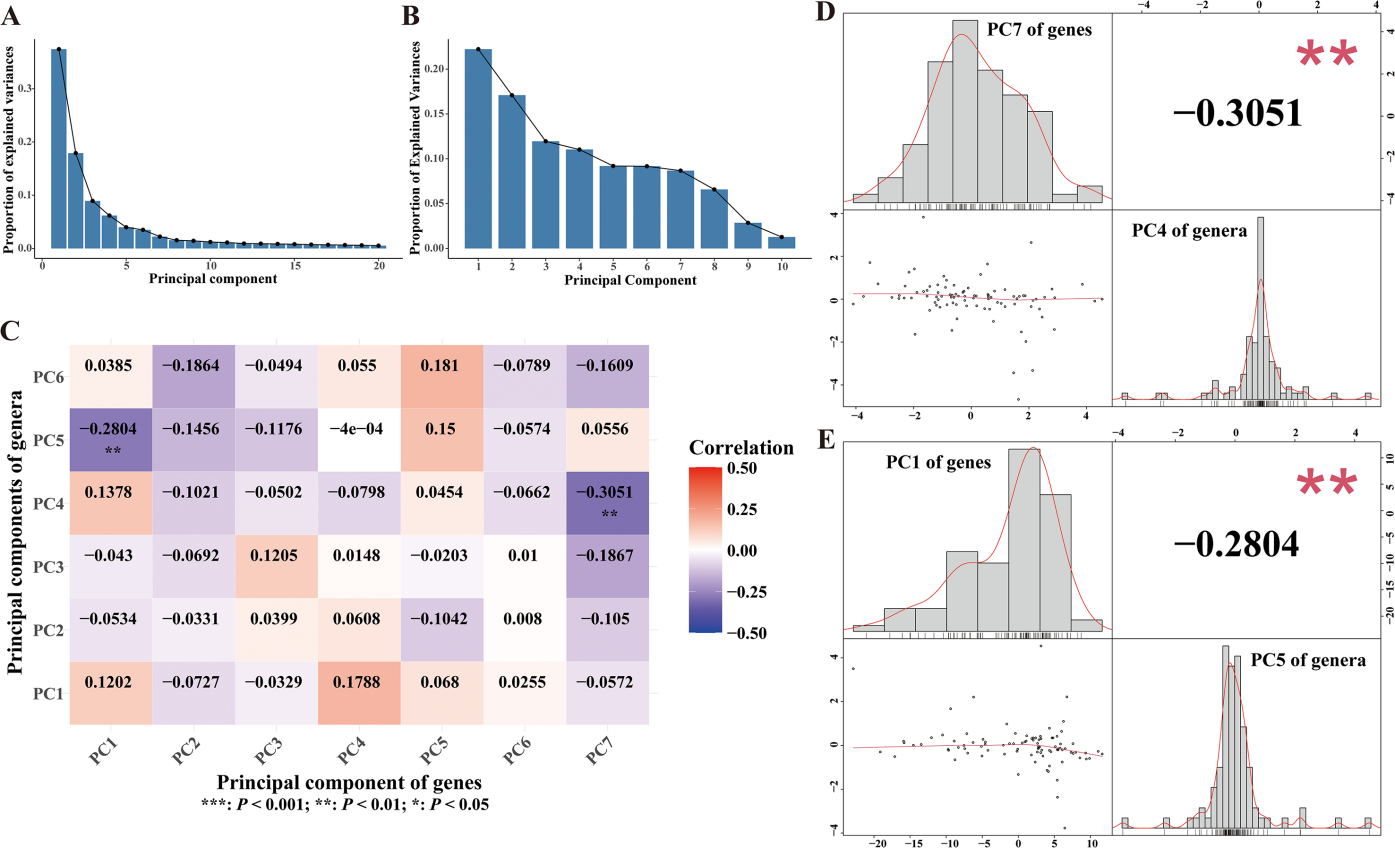
Associations between gut microbiotas and immune signatures. **(A)** Scree plot illustrating explained variances of each principal component of the gene matrix. **(B)** Scree plot illustrating explained variances of each principal component of the genus matrix. **(C)** Heatmap of the correlation coefficients between principal components of gene matrix and principal components of genus matrix. Spearman correlation analysis was performed to calculate the correlation coefficient. **(D, E)** Plots depicted the associations between selected principal component pairs, performed by Spearman analysis. The upper left histogram and lower right histogram showed the distributions of the principal components. The upper right panel indicated the correlation coefficients. Scatter plots were presented in the lower left panels.

For further, we also explored the associations between gut microbiotas and immune signatures disturbed by receptive anal intercourse. To address the high dimensionality and control the false discovery rate, we firstly applied principal component analysis in each dataset. Consequently, seven and six principal components were extracted for gene expression matrix and genus abundance matrix respectively (**Figures 4A, B**). The results of spearman correlation analysis revealed significant negative associations between the first principal component of the gene expression matrix and the fifth principal component of the genus abundance matrix (**Figures 4C, D**). We also observed significant inverse correlations between the seventh principal component of the gene expression matrix and the fourth principal component of the genus abundance matrix (**Figures 4C, E**). These findings revealed that the gut microbiota of MSM were closely associated with immune signatures of PBMCs, indicating that gut microbiome might mediate the effects of sexual behaviors on the activation of immune system.

### 3.5 Single-cell sequencing of PBMC preliminarily elucidates distinct characteristics of CD4^+^ T cells and their association with gut microbiota

**Figure 5:**
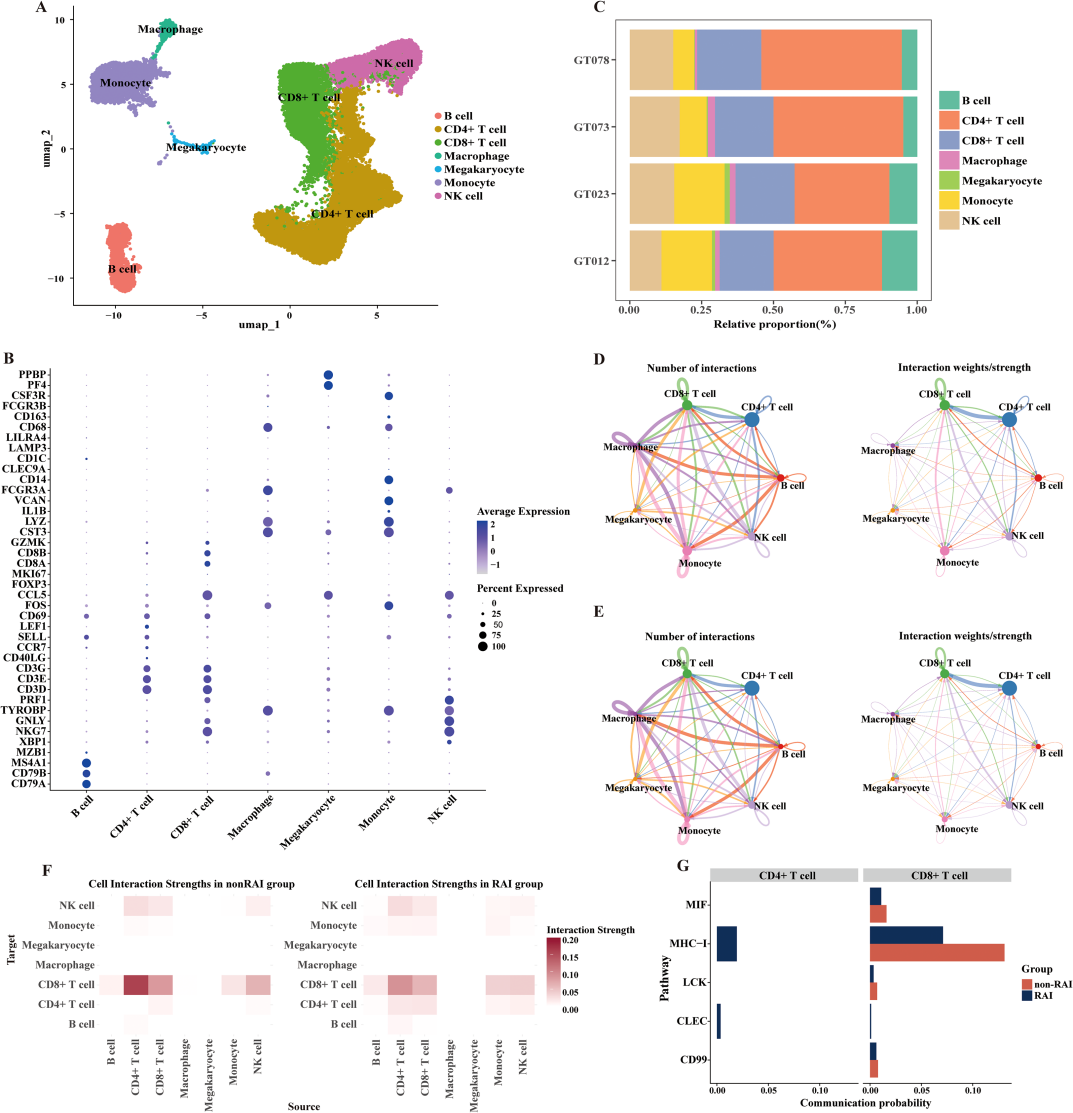
Distinctive activities of CD4+ T cells regarding receptive anal intercourse. **(A)** UMAP plot exhibiting annotated cell types based on known marker genes. **(B)** Dot plot showing the expression of selected marker genes for immune cells. The dot size represents the percentage of cells expressing the selected marker genes, and the dot color represents the mean expression levels of the selected marker genes. **(C)** Bar plot presenting the proportion of different cell types in each sample. GT012 and GT023 belong to the RAI subgroup, while GT073 and GT078 belong to the nonRAI subgroup. **(D, E)** Directed circle plots showing the counts and strength of interactions among cell types in nonRAI subgroup **(D)** and RAI subgroup **(E)** respectively. **(F)** Heatmap depicting the cell interaction strength among cell types in nonRAI subgroup (left) and RAI subgroup (right). **(G)** Bar plots illustrating the self-signal interaction of CD4+ T (left) and outgoing signal from CD4+ T targeting to CD8+ T cells (right) in both groups.

In this section, we further analyzed the heterogeneity of PBMCs in MSM with RAI and MSM without RAI through single-cell transcriptomic sequencing. Since CD4^+^ T cells play as the primary target cells of HIV infection, we specifically focused on the characteristics of CD4^+^ T cells and their associations with RAI-related gut microbiota. PBMCs from four samples were subjected to sequencing for the scRNA analysis, with two samples originating from MSM with RAI and two samples from MSM nonRAI subgroup, respectively (**Supplementary Table S3**). After quality control, we profiled over 27,000 cells, detecting an average of 1,076 genes per cell. Seven immunologic cell types were identified in our data (**Figures 5A, B**), with CD4^+^ T cells being the dominant populations across all samples (**Figure 5C**). We observed an elevated CD8:CD4 ratio in MSM with RAI compared to MSM without RAI. Using the CellChat algorithm, we identified 424 significant communication pairs in the RAI subgroup, while 389 significant communication pairs were observed in the nonRAI subgroup (**Figures 5D, E**). The activities of CD4^+^ T cells in the RAI subgroup were distinctly different from those in the nonRAI subgroup. The strength of communications within CD4^+^ T cells themselves was more active in the RAI subgroup, while no communication was observed in the nonRAI subgroup (**Figure 5F**). Additionally, the outgoing signals of CD4^+^ T cells targeting CD8^+^ T cells were stronger in the nonRAI subgroup than in the RAI subgroup. The communications within CD4^+^ T cells primarily focused on the MHC-I signal pathway and CLEC signal pathway. On the other hand, the MIF signal pathway, MHC-I signal pathway, LCK signal pathway, and CD99 signal pathway were stronger in the outgoing signals of CD4^+^ T cells targeting CD8^+^ T cells in the nonRAI subgroup (**Figure 5G**). These results demonstrated unique characteristics of CD4^+^ T cells in MSM with RAI.

**Figure 6:**
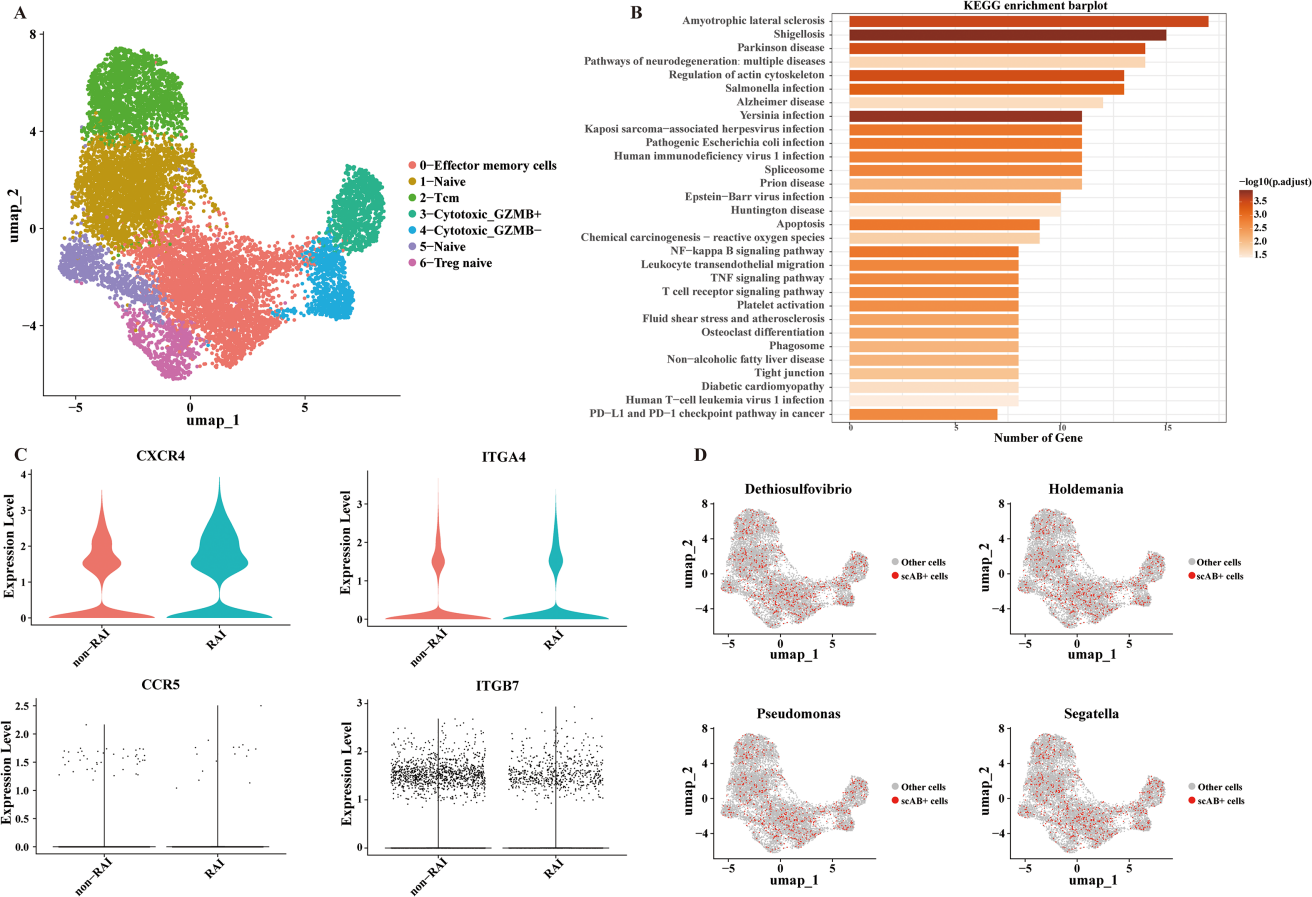
Significant correlations between CD4+ T cells and RAI related genera. **(A)** UMAP plot exhibiting annotated CD4+ T cell subpopulations based on known marker genes. **(B)** Bar plot showing the top 30 enriched KEGG pathways of the differentially expressed genes in CD4+ T cells. **(C)** Violin plot depicting the expression level of markers associated with HIV-1 entry and gut-homing of T cells. **(D)** UMAP plot showing the results of scAB analysis, scAB+ cells represent cells identified to be positively associated with the relative abundance of RAI related genera.

Given the distinct activities of CD4^+^ T cells in relation to RAI and their pivotal role in facilitating HIV entry, we further explored the functions of CD4^+^ T cells and their correlations with genera associated with RAI. We identified four major CD4^+^ T cell subpopulations (**Figure 6A**, **Supplementary Figure S2**): naïve cells (CCR7^+^), cytotoxic cells (NKG7^+^ GNLY^+^), memory cells (IL7R^+^), and regulatory cells (FOXP3^+^). The 191 differentially expressed genes in CD4^+^ T cells between the RAI and nonRAI subgroups (**Supplementary Table S4**) participated in various pathways related to infection, including HIV-1 infection, EB virus infection, and *yersinia* infection (**Figure 6B**). Importantly, we found significantly higher expression of *CXCR4* in the RAI subgroup (P< 0.001), one co-receptor of HIV-1 entry. Additionally, the expression levels of *ITGA4* and *ITGB7*, encoding the gut-homing receptor integrin α4β7, tended to be higher in the RAI subgroup, although not statistically significant (**Figure 6C**). These data indicated that receptive anal intercourse enhanced the gut-homing capacity of CD4^+^ T cells and increased their susceptibility to HIV-1 infection. Finally, based on the scAB algorithm, we leveraged 16S, bulk-seq, and scRNA-seq datasets to illustrate whether RAI-related microbiotas were associated with CD4^+^ T cells. We identified a substantial number of scAB^+^ cells, which were distributed across nearly all CD4^+^ T cell subpopulations (**Figure 6D**). These results are consistent with our findings in the bulk-seq and further emphasize the importance of receptive anal intercourse in shaping gut microbiome composition and modulating the activities of the immune system.

### 3.6 Gut microbiome potentially mediated the effects of sexual behaviors on immune signatures of PBMCs

Building upon the aforementioned findings, our data demonstrate that sexual behaviors induced alterations in gut microbial community and peripheral immune profile, with significant correlations observed between modified microbial species and specific immunological signatures. Furthermore, we utilized mediation analysis to investigate whether gut microbiotas acted as potential mediator in the relationships between sexual behaviors to immune system modulation. Based on the BayesPrism algorithm, we integrated single-cell results and bulk sequencing information to deconvolute bulk RNA sequencing data into distinct cell types. The proportion of each cell type across all samples was stated in **Supplementary Table S5**. Six sexual behaviors were selected as exposure factors since these behaviors altered the diversities of gut microbiome. CD4^+^ T cell proportion, CD8^+^ T cell proportion, CD4:CD8 ratio and HIV-SI were designated as primary outcome measures. Gut microbiotas which showed significant association with both exposures and outcomes were selected as mediators.

The results of mediation analysis were depicted in **Table 2**. In the mediation model consisting of receptive anal intercourse as the exposure variable, *Bilophila* as the mediators and CD4^+^ T cell proportion as the outcome variable, we discovered statistical mediated effects of *Bilophila* on the association between receptive anal intercourse and CD4^+^ T cell proportion with the mediation effects of 40.55% (*P* = 0.026). For more detail, receptive anal intercourse might increase the CD4^+^ T cell proportion by reducing the abundance of *Bilophila*. We also found statistical evidence that *Xylanibacter* might mediate the effects of illicit drug on the proportions of CD4^+^ and CD8^+^ T cells. Specifically, illicit drug decreased the CD4^+^ T cell proportion and increased the CD8^+^ T cell proportion through downregulating the abundance of *Xylanibacter* (*P =* 0.029, *P* = 0.031 respectively). Furthermore, the BayesPrism algorithm also delineated cell type-specific gene expression profiles across all samples. We developed an HIV susceptibility index based on CXCR4, CCR5, ITGA4, and ITGB7 expression levels within CD4^+^ T cells. Notably, exposure to sexual partners with undocumented HIV status was statistically associated with elevated HIV-SI through upregulating the abundance of *Oscillibacter* (*P* = 0.006). Conversely, group sexual activities and illicit drug use were observed to decrease HIV-SI through decreasing the abundance of *Bifidobacterium* (*P* = 0.012, *P* = 0.02 respectively).

**Table 2:** Mediation analyses of gut microbiotas on the association between sexual behaviors and immune signatures (N = 93).

Exposure	Gut microbiotas	Outcome measures	Indirect effects		Direct effects		Total effects	
			Estimates (95%CI)	P value	Estimates (95%CI)	P value	Estimates (95%CI)	P value
Receptive anal intercourse	Weissella	CD4 proportion	-0.017(-0.228, 0.06)	0.599	-0.184(-0.397, 0.02)	0.11	-0.203(-0.413, 0)	0.054
		CD4:CD8 ratio	-0.042(-0.254, 0.04)	0.32	-0.103(-0.303, 0.11)	0.41	-0.145(-0.359,0.07)	0.2
	Xylanibacter	CD4 proportion	-0.013(-0.165, 0.08)	0.663	-0.186 (-0.391, 0.01)	0.094	-0.199 (-0.406, 0.03)	0.084
		CD8 proportion	0.013(-0.082, 0.18)	0.67	0.167(-0.044, 0.36)	0.2	0.179 (-0.079, 0.39)	0.15
	Bilophila	CD4 proportion	0.071(0.007, 0.16)	**0.026**	-0.247(-0.445, -0.08)	0.006	-0.175 (-0.386, 0.03)	0.082
	Oscillibacter	HIV-SI	-0.045(-0.127, 0.02)	0.168	-0.127 (-0.336, 0.06)	0.218	-0.172(-0.367, 0.02)	0.088
	Romboutsia	HIV-SI	-0.007(-0.062, 0.06)	0.823	-0.171(-0.374, 0.03)	0.1	-0.178 (-0.385, 0.02)	0.098
Number of male partners	Ignavibacterium	CD4 proportion	-0.019(-0.095, 0.01)	0.318	-0.207(-0.413, 0.02)	0.066	-0.226(-0.425, -0.01)	0.04
		HIV-SI	-0.026 (-0.113, 0.01)	0.218	-0.202 (-0.398, 0.02)	0.078	-0.228(-0.426, -0.01)	0.04
HIV^+^ partners	Prevotellamassilia	CD8	-0.091(-0.214, 0.06)	0.19	-0.072(-0.465, 0.29)	0.73	-0.162(-0.501, 0.24)	0.42
	Actinomyces	CD8	0.056(-0.021, 0.14)	0.16	-0.211(-0.524, 0.18)	0.26	-0.155(-0.499, 0.24)	0.43
	Romboutsia	CD4:CD8 ratio	0.055 (-0.017, 0.18)	0.15	0.124(-0.273, 0.48)	0.57	0.179(-0.234, 0.52)	0.38
		HIV-SI	0.047(-0.018, 0.15)	0.168	0.265(-0.088, 0.56)	0.156	0.312(-0.050, 0.58)	0.094
	Oscillibacter	HIV-SI	0.079(0.012, 0.15)	**0.006**	0.217(-0.093, 0.56)	0.202	0.296(-0.022, 0.60)	0.068
Group sex with man	Fusobacterium	CD4 proportion	0.108(-0.009, 0.3)	0.11	0.068(-0.304, 0.43)	0.72	0.175(-0.205, 0.51)	0.35
		HIV-SI	0.129(-0.009, 0.35)	0.086	0.081(-0.314, 0.42)	0.661	0.211 (-0.178, 0.51)	0.272
	Bifidobacterium	HIV-SI	-0.097(-0.183, -0.02)	**0.012**	0.275(-0.057, 0.55)	0.088	0.178(-0.168, 0.51)	0.294
	Hominimerdicola	HIV-SI	-0.001(-0.041, 0.08)	0.98	0.198(-0.168, 0.51)	0.28	0.198(-0.173, 0.52)	0.28
Illicit drug	Prevotellamassilia	CD8 proportion	-0.109(-0.231, 0.06)	0.14	0.060(-0.408, 0.42)	0.84	-0.049(-0.474, 0.38)	0.78
	Xylanibacter	CD4 proportion	-0.036(-0.126, 0.00)	**0.029**	-0.082(-0.484, 0.27)	0.714	-0.118(-0.520, 0.25)	0.501
		CD8 proportion	0.035(0.003, 0.13)	**0.031**	-0.069(-0.483, 0.33)	0.634	-0.034(-0.468, 0.35)	0.963
	Bifidobacterium	HIV-SI	-0.096(-0.179, -0.01)	**0.02**	0.148(-0.282, 0.56)	0.42	0.052(-0.353, 0.51)	0.8
STDs	Romboutsia	CD4:CD8 ratio	0.0856(-0.024, 0.25)	0.14	-0.159(-0.594, 0.26)	0.43	-0.073(-0.514, 0.31)	0.65
		HIV-SI	0.085(-0.016, 0.22)	0.14	-0.138(-0.549, 0.29)	0.45	-0.053(-0.514, 0.36)	0.76
	Ignavibacterium	CD4 proportion	0.071(-0.029, 0.19)	0.22	-0.136(-0.567, 0.26)	0.51	-0.065(-0.504, 0.30)	0.68
		HIV-SI	0.089(-0.032, 0.21)	0.16	-0.138(-0.572, 0.29)	0.48	-0.049(-0.511, 0.35)	0.77
	Merdimonas	HIV-SI	-0.008(-0.083, 0.03)	0.73	-0.042(-0.496, 0.39)	0.81	-0.051(-0.509, 0.38)	0.78

The bold values indicate significant mediation effect with P < 0.05.

## 4 Discussion

To our knowledge, this is the first study to systematically investigate the interactions among sexual behaviors, gut microbiome and immune systems in HIV-negative MSM through a multi-omics approach. Previous studies have revealed that *Prevotella* is the dominant genus in MSM (18), and most of these data were generated from the U.S. and Western European nations. Since the gut microbiome is closely associated with geographic locations (31), it is essential to investigate the gut microbiome of MSM in Western China. Our study showed that the gut microbiome of MSM was prominently characterized by *Segatella*, a novel genus previously classified within the *Prevotella* genus (32). One recent study identified substantial influences of multiple sexual partners on gut microbial diversity (17). Specifically, our data reported that alpha and beta diversity were primarily altered by receptive anal intercourse. Meanwhile multiple male sexual partners were also observed to increase the alpha diversity of gut microbiome in our study. Consistent with our results, I. Vujkovic-Cvijin and colleagues demonstrated that receptive anal intercourse was associated with unique gut microbiota signatures in both MSM and women, regardless of condom use, characterized by enrichment of the *Prevotella* genus (6). Our study further emphasized the important role of receptive anal intercourse in perturbing the gut microbial composition. However, MSM in the RAI group engaged in both receptive and insertive anal intercourse in our study. Future studies are expected to enroll MSM who engaged exclusively in receptive anal intercourse for better exploring the association between anal intercourse and gut microbiome. Additionally, we found that receptive anal intercourse altered immune signatures at both the bulk-seq and scRNA-seq levels, which were enriched for HIV infection pathways. These immune signature alterations were significantly correlated with the gut microbiome. Furthermore, the results of mediation analysis suggested potential mediation effects of gut microbiome on the associations between sexual behaviors and CD4^+^ T cell proportions and HIV susceptibility. These findings support our hypothesis that high-risk sexual behaviors increase susceptibility to HIV infection through gut microbiome dysbiosis. While, only 4 samples were included in the scRNA analysis, thus these observations require validation in a larger cohort.

Mankind has been working for decades towards the goal of ending AIDS, and the worldwide new HIV infection are decreasing in recent years. Globally, the proportion of MSM in new HIV infections has increased to 20% in 2022, twice that of 2010 (33). The high prevalence of HIV infection in MSM is often attributed to behavioral factors, such as condomless sex, multiple sex partners, inappropriate anal douching, and engaging in receptive anal intercourse (28, 34–36). However, less is known about the biological factors. Analyzing the gut microbiome of MSM offers novel perspectives for the prophylaxis of HIV. The gut microbiome dysbiosis caused by high-risk sexual behaviors might persistently exist and act as a risk factor for HIV acquisition. Jennifer A. Fulcher et al. conducted a longitudinal study and indicated that gut microbiome differences existed in seropositive participants compared with matched controls before seroconversion. In contrast, after acute HIV infection, the gut microbiome changes were subtle (2). A former retrospective study performed by Yue Chen et al. also stated similar results and indicated that gut microbiome changes in HIV-negative MSM were associated with increased susceptibility to HIV-1 infection (1). Brent E. Palmer conducted a series of researches and proposed that MSM-associated gut microbiome enhanced HIV infection through elevating the frequency of HIV-targeted cells in the colon and promoting T cell homing to the gut from peripheral blood (23). Interestingly, one of our previous studies also suggested causal correlations between gut microbiome and HIV infection based on a two-sample Mendelian randomization research, further demonstrating their close correlations (37). Our study identified significant gut microbiome alterations associated with receptive anal intercourse, which might partially elucidate the higher risk of HIV infection among MSM engaged in receptive anal intercourse.

HIV infection heavily relies on the immune system. In the transition process of the virus, HIV-1 envelope glycoprotein interacts with the primary receptor CD4 and coreceptor CCR5 or CXCR4 to enter the host cells and produce the infection (38). The gut mucosa acts as the primary site of HIV entry in MSM, and the immune environment of the gut appears to be important for HIV acquisition and transmission. The integrin α4β7 induces the homing of immune cells to the large and small intestine by binding to its ligand MAdCAM-1 (30). Aftab A. Ansari et al. developed an antibody against α4β7, and demonstrated that administration of α4β7 antibody before and during repeated low-dose intravaginal simian immunodeficiency virus (SIV) challenge of rhesus macaques significantly protected the animals from SIV transmission (39). In SIV-infected rhesus macaques, antiretroviral therapy combined with α4β7 antibody contributed to effectively controlling viremia (40). In humans, the expression level of α4β7 in peripheral blood CD4^+^ T cells predicted higher rates of HIV acquisition and higher set point viral load in South African women (41). Our data showed that the expression of *ITGA4* and *ITGB7* in peripheral blood CD4^+^ T cells tended to be higher in the RAI group, indicating a higher gut-homing propensity of CD4^+^ T cells in MSM engaged in receptive anal intercourse. Moreover, we found that *CXCR4* was significantly higher expressed in CD4^+^ T cells in the RAI group compared with the non-RAI group, facilitating these cells higher susceptibility to HIV. These clues suggest that receptive anal intercourse increases the possibility of CD4^+^ T cell migration to the gut and interaction with HIV. Notably, gut microbiotas were significantly associated with CD4^+^ T cells according to our results, further emphasizing the regulatory role of the gut microbiome in the immune system.

The interactions between the gut microbiome and the immune system are likely mediated by gut microbiota-derived metabolites (42). Gut microbiota-derived metabolites can be transported to various organs of the human body and have been shown to be significantly associated with pro-inflammatory factors in the brains of Alzheimer’s mice (43). Specifically, short-chain fatty acids (SCFAs) are key metabolic by-products synthesized by gut microbiota. Researchers have found that SCFAs can activate the GPR41 and GPR43 signaling pathways, which are associated with metabolic diseases and inflammatory responses (44). SCFAs also participate in maintaining intestinal homeostasis by regulating Th1 cell function (45). Recently, Felix F. Krause et al. found that *Clostridium* sp*orogenes*-derived indole-3-pro-pionic acid (IPA) reduced IL-17A protein expression, while branched-chain fatty acids (BCFAs) and SCFAs improved the activity of regulatory T cells and increased the level of IL-22 (46). In HIV-infected patients, SCFAs also play an important role in maintaining immune homeostasis. HIV-1 infected patients have lower relative abundance of butyrate-producing bacterial species, and intestinal cells cultured with butyrate showed suppressed T cell activations and HIV-1 infection levels (47). In our analysis, we found that having group sex with men could decrease the abundance of *Bifidobacterium*. Interestingly, *Bifidobacterium* could produce SCFAs (48). The reduction of SCFAs production caused by *Bifidobacterium* depletion might be the potential mechanism affecting HIV susceptibility. For future studies, metabolomic research and animal models could be applied to better explain the interactions between the gut microbiome and immune activities in MSM.

There are several limitations in our study. Firstly, data on sexual behaviors were collected by self-reported questionnaires, which may introduce bias if participants provided unreliable responses. Since sexual behavior information is private, participants may be afraid of privacy disclosure. This bias could be mitigated if researchers devise more effective and safer approaches to collect such information. Additionally, this is a cross-sectional study, which cannot clarify the causal relationships between gut microbiome dysbiosis and increased susceptibility to HIV infection, although our mediation analysis revealed potential causal effects of gut microbiome on the associations between sexual behaviors and HIV susceptibility. Longitudinal studies are still needed to illustrate the gut microbiome patterns of HIV-negative MSM and to demonstrate the causal relationships between gut microbiome dysbiosis and HIV infection. Thirdly, our study investigated the gut microbiome through 16S rRNA sequencing which provides limited taxonomic resolution (often to the genus level) and does not directly yield functional information. We encourage future studies apply shotgun metagenomics to investigate gut microbiome in MSM population. Furthermore, our study only recruited MSM in Chongqing city of China, it remains to be elucidated whether our conclusions could be applied in other populations.

## 5 Conclusions

Our study revealed profound impacts of sexual behaviors, especially receptive anal intercourse, on the gut microbiome composition and systemic immune activations in HIV-negative MSM. Receptive anal intercourse increased the diversity of the gut microbiome in HIV-negative MSM. Moreover, we suggested close correlations between gut microbiota and systemic immune signatures. Critically, mediation analysis revealed that *Bilophila* mediated the effects of receptive anal intercourse on elevated CD4^+^ T cell proportions (*P* = 0.026), and *Oscillibacter* mediated the association between exposure to partners of undocumented HIV status and heightened HIV susceptibility (*P* = 0.006). These findings directly link gut microbiota to sexual behavior-driven immune dysregulation. Our multi-omics approach highlights the gut microbiome as a pivotal mediator between high-risk sexual behaviors and HIV susceptibility. Longitudinal studies are expected to validate causality and explore microbiota-targeted interventions for HIV prevention. This work advances the understanding of biological pathways underlying HIV acquisition in MSM and underscores the gut-immune interface as a novel preventive target.

## Data Availability

The raw sequencing data of this study have been deposited in the NCBI Sequence Read Archive. The accession numbers for these datasets are as follows: 16S rRNA gene sequencing data: PRJNA1191636. Bulk transcriptome sequencing data: PRJNA1192389. Single-cell transcriptome sequencing data: PRJNA1193218.

## Supplementary material

Supplementary Figure 1 Comparisons of alpha and beta diversities across different sexual behaviors. **(A-D)** Box plots representing the alpha diversities across sexual behavioral subgroups, including HIV+ partners, group sex with man, STDs and illicit drug. Shannon, Simpson, Ace and Richness indices were calculated to evaluate the alpha diversities. **(E-H)** Principal-coordinate analysis (PCoA) based on Bray-Curtis distance indicated no differences of beta diversity across these sexual behaviors.

Supplementary Figure 2 Dot plot showing the expression of selected marker genes in CD4+ T cells. The dot size represents the percentage of cells expressing the selected marker genes, and the dot color represents the mean expression levels of the selected marker genes.
