# Supplementary figures and images for: Gut microbiome as a potential mediator linking sexual behaviors to immune profiles in HIV-negative men who have sex with men: a multi-omics study
Source: Front Immunol. 2025 Oct 16;16:1659556. doi: 10.3389/fimmu.2025.1659556 (PMC12571833; doi:10.3389/fimmu.2025.1659556)

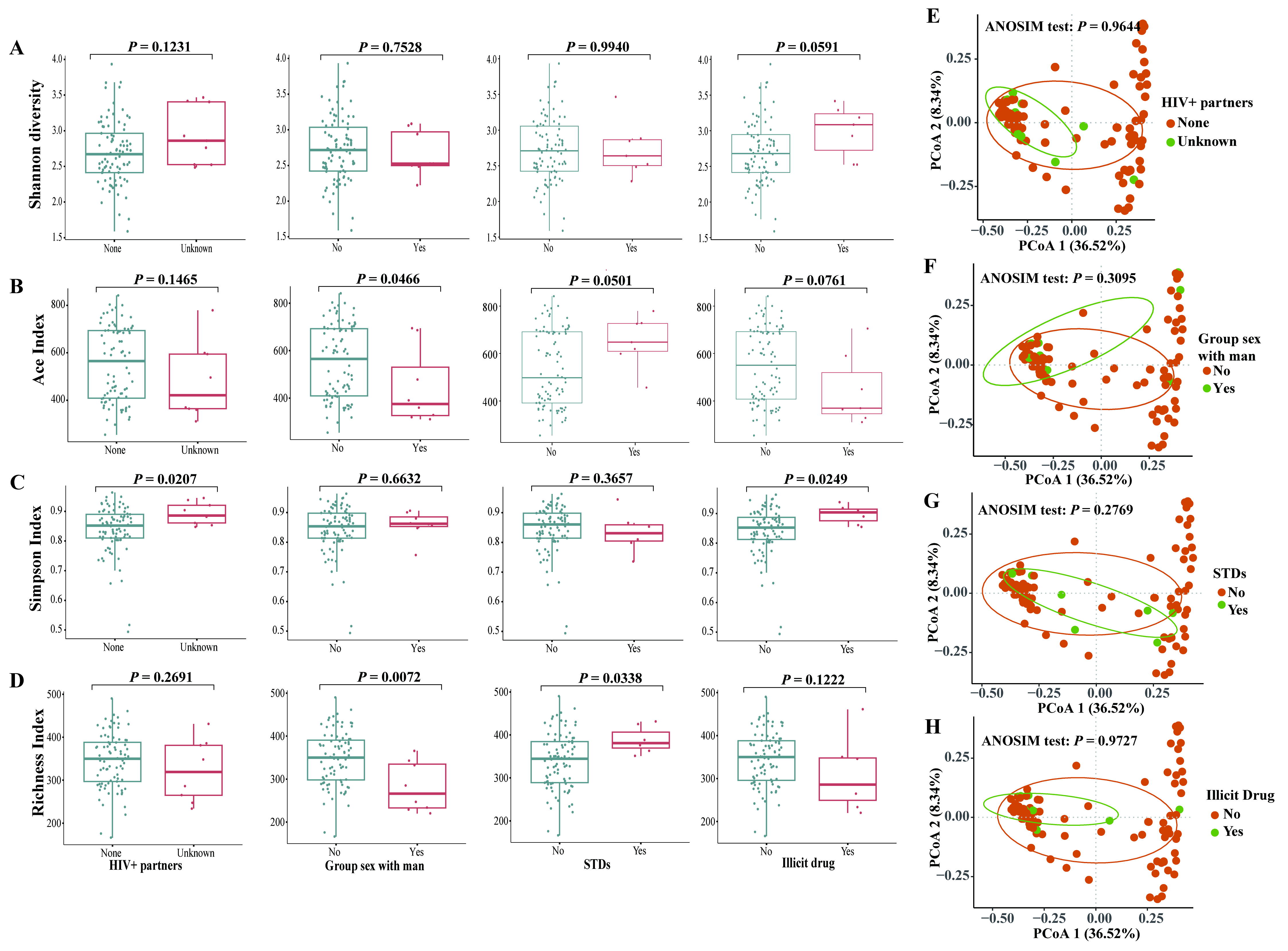

Supplement: Supplementary Figure 1 — Comparisons of alpha and beta diversities across different sexual behaviors. (A-D) Box plots representing the alpha diversities across sexual behavioral subgroups, including HIV+ partners, group sex with man, STDs and illicit drug. Shannon, Simpson, Ace and Richness indices were calculated to evaluate the alpha diversities. (E-H) Principal-coordinate analysis (PCoA) based on Bray-Curtis distance indicated no differences of beta diversity across these sexual behaviors. [file Image1.tif]

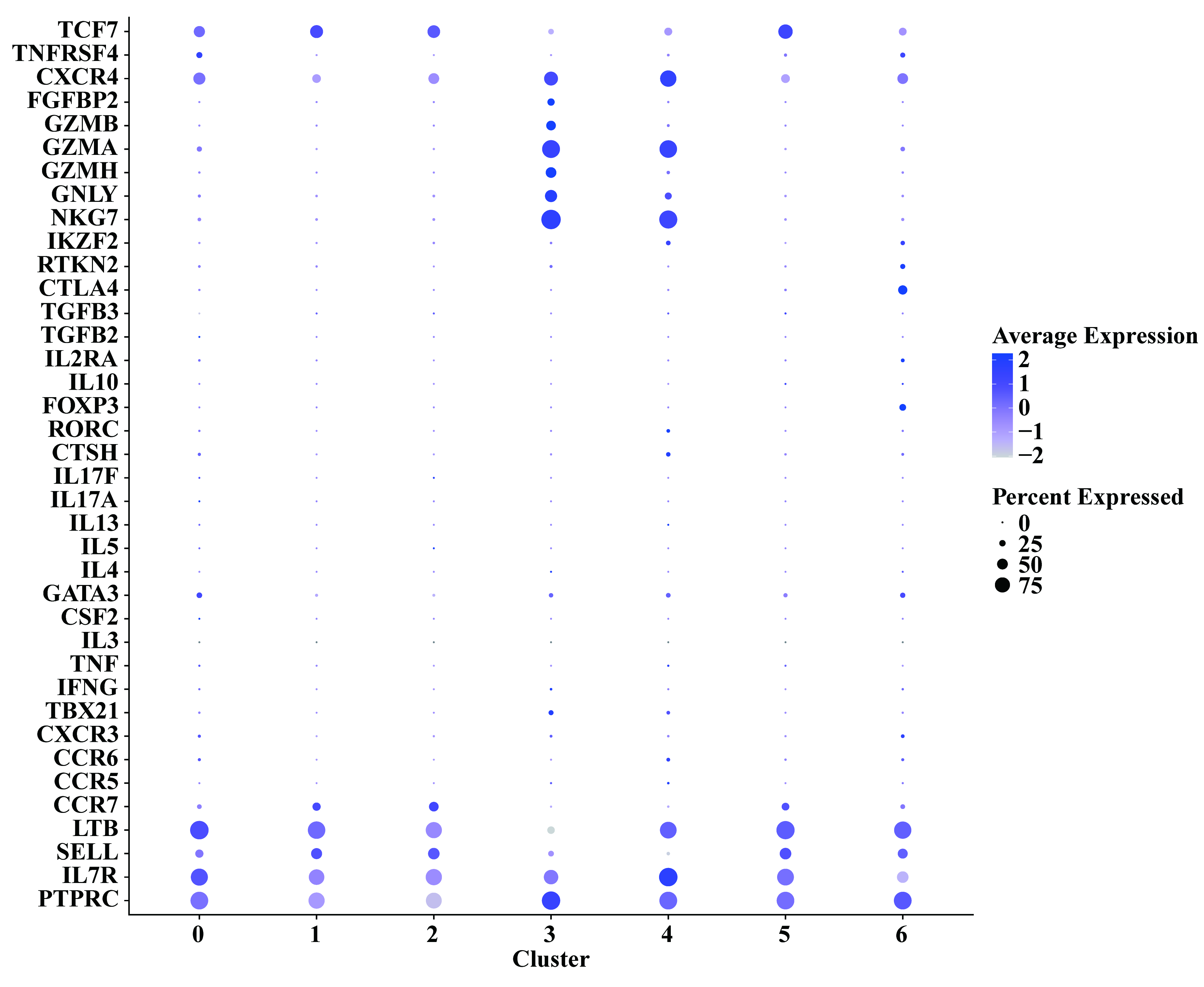

Supplement: Supplementary Figure 2 — Dot plot showing the expression of selected marker genes in CD4+ T cells. The dot size represents the percentage of cells expressing the selected marker genes, and the dot color represents the mean expression levels of the selected marker genes. [file Image2.tif]
